# A Novel Fluoride Route for the Synthesis of Aluminosilicate Nanotubes

**DOI:** 10.3390/nano3010117

**Published:** 2013-02-06

**Authors:** Atika Chemmi, Jocelyne Brendlé, Claire Marichal, Bénédicte Lebeau

**Affiliations:** Equipe Matériaux à Porosité Controlée (MPC), Institut de Science des Matériaux de Mulhouse (IS2M), CNRS UMR 7361, Université de Haute Alsace (UHA), ENSCMu, 3b rue Alfred Werner, 68093 Mulhouse Cedex, France; E-Mails: atika.chemmi@uha.fr (A.C.); claire.marichal@uha.fr (C.M.); benedicte.lebeau@uha.fr (B.L.)

**Keywords:** aluminosilicate nanotube, imogolite, fluoride route, synthesis

## Abstract

In this work we present a novel method for synthesis of aluminosilicate nanotubes: the fluoride route. F-containing imogolite (F-IMO) exhibits an improved crystallization rate and improved yield. The structure of F-IMO was investigated and compared with F-free imogolite (IMO) by means of X-ray diffraction (XRD) and Fourier transformed infrared spectroscopy (FTIR) confirming imogolite structure. Solid state nuclear magnetic resonance (NMR) analyses show an increased crystallization rate for F-IMO and confirm the incorporation of fluorine ion in the structure.

## 1. Introduction

Imogolite is a natural mineral observed for the first time by Aomine *et al.* in 1962 [1]. It consists of a single-walled aluminosilicate nanotube with inner and outer diameters of 1 and 2 nm, respectively, and a length ranging from a few tens to several hundreds of nanometers. The tube walls are composed of a curved gibbsite (Al(OH)_3_) layer on the outer surface and silicate tetrahedra linked to six aluminum octahedra inside the tube [2]. The empirical formula of imogolite is (HO)_3_Al_2_O_3_SiOH. Imogolite was first prepared by Farmer *et al*. in 1977 by coprecipitation of aluminum and silicon monomer with millimolar initial concentrations at 95 °C [3]. Due to its hollow tubular structure, imogolite is nowadays considered as a competent substitution for carbon nanotubes. However imogolite differs from carbon nanotube by its hydrophilic surface which can be easily modified to obtain hybrid materials [4]. Thus, several applications of this material have been reported including catalysis [5], gas storage [6], adsorption [7] and heat exchange [8]. Several synthesis strategies have been developed recently in order to optimize imogolite synthesis by varying some parameters such as nature of reactants, temperature and growth phase duration, desalination process, recovering and drying. Nevertheless it is still difficult to produce a high yield of imogolite. Furthermore, some advances have been made about determination of the formation mechanism [9,10,11,12,13,14,15], but some problems remain to be overcome for industrial development of imogolite.

From these considerations, we have investigated the possibility to use the fluoride route for the synthesis of imogolite. Indeed, the fluoride route is extensively used for synthesis of aluminosilicate, e.g., zeolites and clays such as beidellites, montmorillonites and saponites [16,17]. The F^−^ fluoride ion acts as a mineralizing agent such as the hydroxide anion because of its similar charge and size. The fluoride ions used in small amounts, were proven to decrease crystallization times and increase the crystallinity of the solids. Moreover the substitution of hydroxyl groups by fluoride ions in the structure allows increasing the thermal stability of the material. In addition, the ^19^F nucleus, thanks to its ½ nuclear spin and 100% natural abundance, is an interesting probe for structural investigations by solid-state NMR. To the best of our knowledge, this route has never been explored for the imogolite synthesis.

## 2. Experimental Section

### 2.1. Synthesis of F-Containing Imogolite

In this work, imogolite nanotube was synthesized in fluoride medium by coupling two protocoles: the imogolite synthesis pathway proposed by Suzuki *et al.* [18] and the fluoride route developed for clays synthesis by Huve *et al.* [19]. Suzuki protocol has been selected to avoid use of perchlorates.

F-containing imogolite (F-IMO) was synthesized in an acidic fluoride medium via sol-gel process using hydrogels with the following molar composition: 1 SiO_2_: 3.5 NaOH: 2.5 Al_2_O_3_: 0.1 HF: 111.1 H_2_O. 

In a typical synthetic procedure, an aqueous solution of 150 mM of aluminum chloride (AlCl_3_·6H_2_O, Fluka) was mixed with an aqueous solution of 60 mM of sodium orthosilicate (Na_4_SiO_4,_ Alfa Aesar) where the Al and Si ratio was 2.5. Then a 1 M sodium hydroxide (NaOH, Riedel-de-Haen) solution was added dropwise until the pH of the solution reached 6. Next, the desalination process was carried out three times using centrifugation and the collected solid was then dispersed in 2 L of distilled water. At the end of the dispersion, hydrofluoride acid HF (5 wt.%) was added and left under stirring for 10 min. The pH of the suspension initially about 5 was lowered down to 4–4.5 by dropwise addition of 1 M hydrochloric acid (HCl, Carlo Erba). The mixture was stirred for 10 min at room temperature, transferred into a closed polypropylene flask and aged at 98 °C for 7 days. After cooling to room temperature, a limpid solution is obtained (pH = 3.5–4). A solution of ammonia (25 wt.%) was added until the pH reached 8. The solid was recovered by centrifugation and washed with distilled water. The gel collected was redispersed in distilled water and lyophilized. The obtained solid (F-IMO) was characterized by using XRD, FTIR, TEM, NMR and elemental analysis. A reference imogolite (IMO) was also synthesized according to the Suzuki procedure [18] without introduction of HF.

### 2.2. Characterization

X-ray diffraction (XRD) patterns were recorded at room temperature on a powder PANalytical X’PERT PRO diffractometer (PANalytical, Limeil-Brevannes, France), equipped with Cu anode (λκ_α_ = 1.5418 Å, 3° < 2θ < 70°, 0.02°/s).

TEM images were collected on a Philips CM200 microscope equipped with a LaB_6_ filament. The accelerating voltage was 200 kV. The samples were prepared by depositing several drops of diluted suspension onto Cu grids coated with a thin (5 nm) holey carbon film.

Fourier Transformed Infrared spectra (FTIR) were carried out using Bruker Equinox 55 spectrometer (Bruker Optics, Marne la Vallée, France). The samples were mounted as KBr disks and routinely recorded using resolution of 4.0 cm^−1^ and 200 scans between 4000 and 200 cm^−1^.

Elemental analysis of F-IMO was performed for the following elements: Al, Si, H and F by the Service Central d’Analyse du CNRS in Solaize, France.

^1^H-^29^Si solid-state CP-MAS NMR spectra were recorded on a Bruker AVANCE II 300WB spectrometer (B_0_ = 7.1 T) operating at 59.59 MHz, with a pulse duration of 3.5 μs corresponding to a flip angle of π/2, a contact time of 4 ms and a recycle delay of 4 s. Samples were packed in a 7 mm cylindrical zirconia rotor and spun at a spinning frequency of 4 kHz. ^29^Si chemical shifts were referenced to tetramethylsilane (TMS). ^27^Al and ^19^F MAS NMR spectra were recorded on a Bruker Avance II 400WB spectrometer (B_0_ = 9.4 T) operating at 104.2 and 376.05 MHz, respectively. ^27^Al chemical shifts were given relative to an aqueous solution of aluminum nitrate (Al(NO_3_)_3_).

^27^Al MAS NMR experiments were recorded using a 4 mm cylindrical zirconia rotor and spun at a spinning frequency of 14 kHz. Typical acquisition parameters included 0.6 μs corresponding to a flip angle of π/12 pulse and 0.5 s recycle delay. ^19^F MAS NMR experiments were recorded using a 2.5 mm cylindrical zirconia rotor, at a spinning frequency of 28 kHz. Typical acquisition parameters included 4 μs corresponding to a flip angle of π/2 pulse and 30 s recycle delay. Note that the low amount of fluorine in the sample prevents T_1_ measurements. ^19^F chemicals shifts were given relative to trichloro-fluoro-methane (CFCl_3_).

Decompositions of the NMR spectra to extract the proportion of the corresponding species were performed with the DMfit software [20].

## 3. Results and Discussion

**Figure 1:**
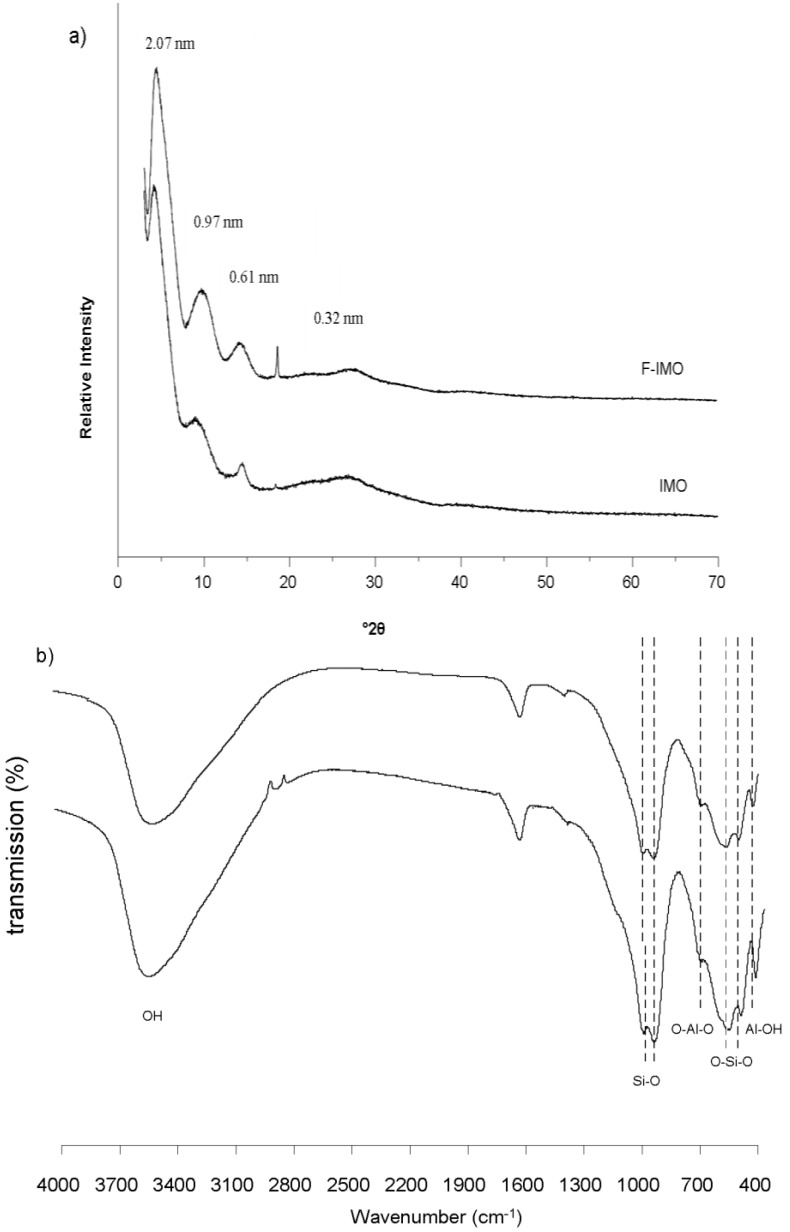
(**a**) XRD pattern and (**b**) FTIR spectra of F-containing imogolite (F-IMO) and reference imogolite (IMO).

The yield of the freeze-dried products was 64% for F-IMO against 44% for IMO. The XRD diffraction patterns of F-IMO and IMO are presented in Figure 1(a). Both diffractograms show four broad reflections at 4.2°2θ (2.07 nm), 9.1°2θ (0.97 nm), 14.3°2θ (0.61 nm) and 26.7°2θ (0.32 nm) characteristic of imogolite structure [2,13,21]. The most intense reflection at 4.2°2θ is attributed to the (100) plane of the overall structure, and is related to the diameter of the nanotubes (2.07 nm) [2]. The reflection at 9.1°2θ (0.97 nm) is assigned to the (001) plane and characterizes the fiber structure [9]. It is noteworthy that the sharp reflection at 18.6°2θ (0.47 nm) clearly observed for F-IMO and barely for IMO is not yet assigned.

Figure 1(b) shows similar FTIR spectra of the F-IMO and IMO. The bands characteristic of imogolite appear at 992, 953, 697, 562, 512 and 425 cm^−1^. The band doublet observed at 992 and 953 cm^−1^ corresponds to the Si-O stretching vibration, which are specific for tubular structure [22]. The bands at 697 and 425 cm^−1^ are attributed to O-Al-O and Al-OH vibrations. The bands observed at 562 and 512 cm^−1^ are related to O-Si-O vibrations. The FTIR results are consistent with the formation of imogolite in fluoride medium and confirm XRD results.

**Figure 2:**
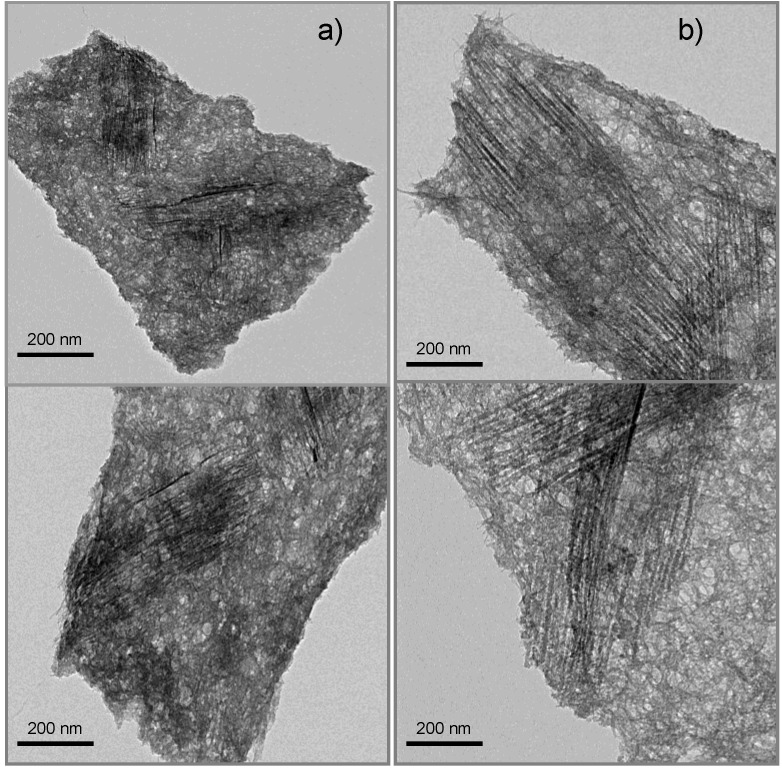
TEM micrographs of F-IMO (**a**) and IMO (**b**).

The morphology of F-IMO was observed by TEM. The micrographs in Figure 2 show that, like IMO, F-IMO forms well-distinguished individual fibers. The fibers are aligned in bundles. A spiderweb-like structure was also observed for both samples. The insufficient dilution of the sample prevents an accurate determination of the length and the diameter but by using Image J software the diameter was evaluated. No significant difference concerning the diameter value is observed between F-IMO and IMO. The average outer diameter is roughly 2.4 nm for both samples, which is slightly higher than the one estimated from XRD results. 

**Figure 3:**
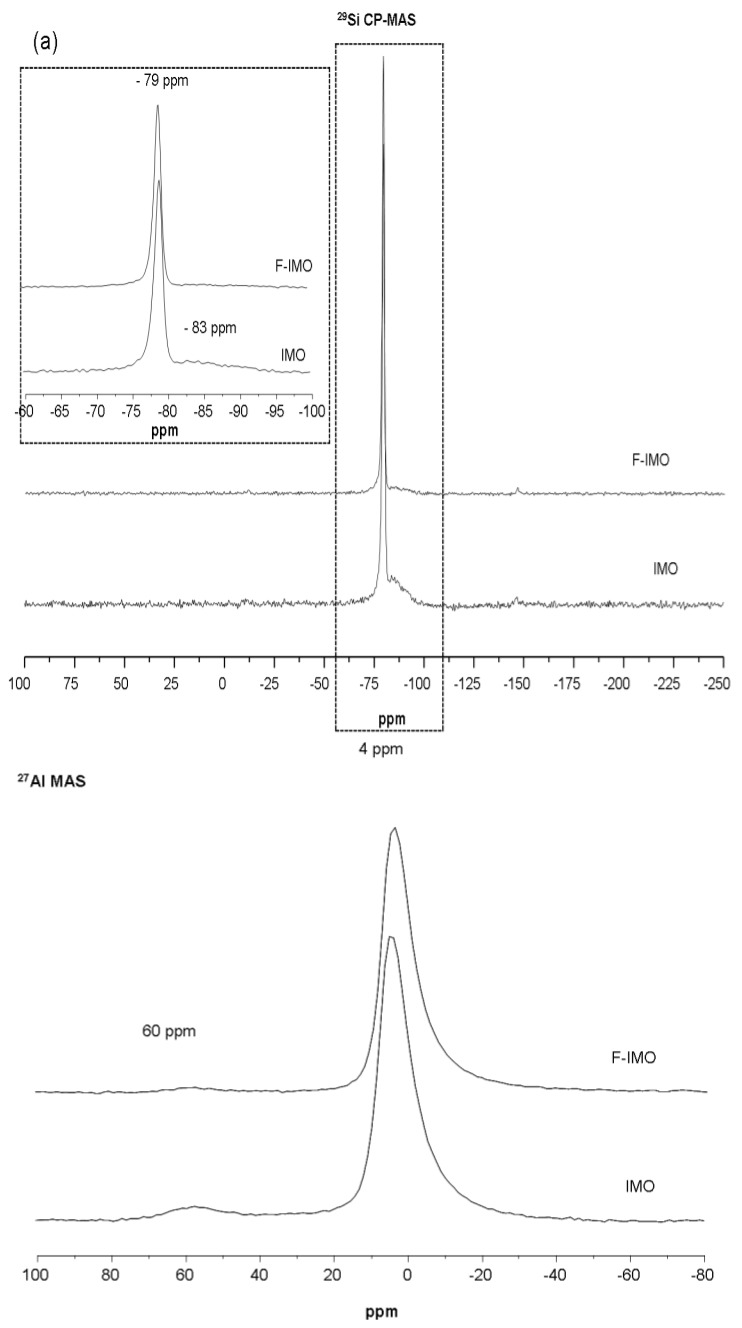
(**a**) ^29^Si CP-MAS NMR spectra of F-containing imogolite and reference material imogolite; (**b**) ^27^Al MAS NMR spectra of F-containing imogolite and reference material imogolite.

The local environment of Si, Al and F in F-IMO was probed by solid-state NMR spectroscopy. The ^29^Si CP-MAS NMR spectra of F-IMO and IMO samples are displayed in Figure 3(a). CP-MAS usually do not allow a quantitative approach but is used to improve the signal to noise ratio and save spectrometer time. Nevertheless, the ^1^H decoupled ^29^Si MAS NMR spectrum recorded for the IMO sample lead to the same intensity ratio for all resonances. Therefore, in this particular case, it is possible to compare both samples. For both sample a main resonance at −79 ppm is detected and assigned to silanol groups connected to 6 Al through oxygen atoms, as expected for imogolite structure [23]. The width at half height, in the case of the IMO sample is 73 Hz instead of 60 Hz for the F-IMO sample. The broadening of the line is often related to a distribution of bond lengths or angles suggesting that imogolite prepared in fluoride medium seems to be better organized on a local point of view. Furthermore, additional weak and very broad resonances are detected on both ^29^Si CP-MAS NMR spectra (Figure 3 inset). Decomposition of the lines reveals that these broad components appear between −83 ppm and −95 ppm and account for 10% and 32% of the total signal in F-IMO and IMO samples, respectively. Those components are assigned to less ordered silicon environments indicating the presence of poorly crystallized species called protoimogolite or/and allophone [21,22], but could also correspond to structural defects present in the imogolite walls as recently demonstrated by Yucelen *et al.* [24]. At the stage of the study it is not possible to discriminate between both hypotheses but whatever the assignment of these resonances the presence of fluorine in the synthesis medium appears to decrease the proportion of unwanted species. ^27^Al MAS NMR spectra of both samples are shown in Figure 3(b). Two resonances are observed at 4 ppm and 60 ppm corresponding to the octahedral and tetrahedral aluminum, respectively. This tetrahedral aluminum could correspond to either transient phase formed in variable amount during imogolite synthesis or to aluminum sites at the end of the nanotubes as suggested by Nair *et al.* [14]. It is interesting to note that, the proportion of tetrahedral aluminum is negligible (2% of the total signal) when the synthesis was performed in fluoride medium instead of 6% without fluorine. Both ^27^Al MAS and ^29^Si CP-MAS NMR suggest a lower amount of these poorly crystalline species or defect sites when the fluoride route is used. As reported for other minerals, the synthesis in the presence of fluorine allows increasing the crystallization rate [19]. 

Elemental analysis of F-IMO was performed for the following elements: Al, Si, H and F. The results are listed in Table 1. It should be noted that a small amount of fluorine was incorporated in F-IMO (less than 1%). Based on these results and on ^27^Al MAS and ^29^Si CP-MAS solid state NMR data, the following formula can be given for F-IMO: (HO)_3_O_3_Al_2_(Si_0.9_Al_0.1_)(F_0.08_OH_0.92_). It is noteworthy that there is slightly more Al in the F-IMO than in classical imogolite. However uncertainty of element analysis is about 5%.

**Table 1:** Elemental analysis of F-IMO.

Elemental analysis (wt.%)			
Al	Si	F	H
23.00	9.89	0.63	3.72

**Figure 4:**
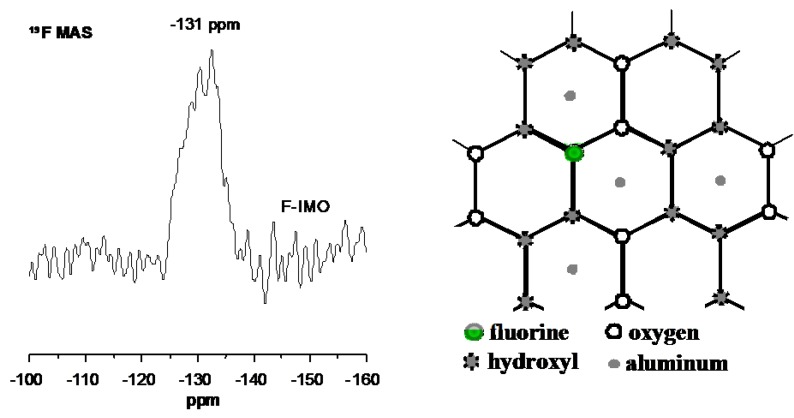
^19^F MAS NMR spectrum of F-containing imogolite and in plane structure of the octahedrallayer showing the local environment of fluorine ion.

^19^F MAS NMR spectra of F-IMO displayed in Figure 4 shows a broad resonance at about −131 ppm confirming the presence of fluorine. The very low signal to noise ratio of the spectrum suggests that only a weak amount of fluorine has been incorporated in the structure, in agreement with chemical analysis (<1 wt.%). The observed chemical shift value (−131 ppm) is close to the one observed for beidellite, a dioctahedral phyllosilicate prepared in fluoride medium [19]. In that case, a ^19^F signal at −132 ppm was detected and assigned to fluorine atom linked to Al-Al-□ in octahedral sheet (where □ represents a vacancy). A similar atom arrangement is also probably present in F-IMO. The broadness of the line suggests a distribution of environments for the fluorine.

## 4. Conclusions

In conclusion, aluminosilicate nanotubes were successfully obtained by a new synthesis method using fluoride medium with a yield as high as 64% (against 44% in absence of fluorine). According to solid state NMR, a lower amount of poorly crystalline species or defects sites are observed when the fluoride route is used. Studies are in progress to fine-tune the different parameters governing the synthesis and to explore the mechanism of formation taking advantage of the presence of fluorine.
